# Complete Genome Sequence of Bifidobacterium longum Strain Jih1, Isolated from Human Feces

**DOI:** 10.1128/MRA.00319-20

**Published:** 2020-05-28

**Authors:** Hikaru Inoue, Sayaka Shibata, Konosuke Ii, Joe Inoue, Shinji Fukuda, Kazuharu Arakawa

**Affiliations:** a Institute for Advanced Biosciences, Keio University, Tsuruoka, Japan; b Systems Biology Program, Graduate School of Media and Governance, Keio University, Fujisawa, Japan; c Intestinal Microbiota Project, Kanagawa Institute of Industrial Science and Technology, Kanagawa, Japan; d Faculty of Pharmacy, Graduate School of Pharmaceutical Science, Keio University, Tokyo, Japan; e Faculty of Pharmacy, Graduate School of Pharmaceutical Sciences, Kumamoto University, Kumamoto, Japan; f Transborder Medical Research Center, University of Tsukuba, Ibaraki, Japan; g Faculty of Environment and Information Studies, Keio University, Fujisawa, Japan; University of Maryland School of Medicine

## Abstract

We report the complete genome sequence of Bifidobacterium longum strain Jih1, isolated from human feces. The assembled genome comprised one circular chromosome of 2.37 Mb. The chromosome harbors 1,941 protein-coding genes.

## ANNOUNCEMENT

Bifidobacterium longum is a Gram-positive, catalase-negative bacterium in the human gut microbiota (1, 2). *Bifidobacterium* dominates in healthy breastfed infants, and the gut microbiota composition of infants can affect long-term human health (3). Previous studies have indicated that probiotic bifidobacteria protect mice against Shiga toxin-producing Escherichia coli O157:H7 lethal infection (4–6). In this study, strain Jih1 was isolated from healthy human feces using TOS propionate agar medium (Yakult) and grown in 5 ml of GAM medium (Nissui). The species of the isolate was determined by a BLAST search of the 16S rRNA gene sequence, which showed 99% identity with B. longum subsp. *infantis* strain ATCC 15697. The incubation was carried out overnight at 37°C in an anaerobic jar containing a carbon dioxide-generating sachet. Genomic DNA was extracted with the Genomic-tip 20/G (Qiagen). The sequencing library for long reads was prepared using a rapid barcoding kit (SQK-RAB004), sequenced in a flow cell (FLO-MINI106) with a GridION device (Oxford Nanopore Technologies), and base called with GridION version 19.10.2 software in high-accuracy mode. Illumina sequencing was performed for error correction using a HyperPlus kit (Kapa Biosystems) for library preparation, and the genome was sequenced on a NextSeq 500 sequencer using 75-cycle high-output mode (Illumina).

A total of 341,428 long reads (*N*_50_, 7.2 kbp) were obtained. After filtering for length over 15,000 bp to obtain around 100× coverage, the reads were assembled using Canu version 1.8 (7). The genome was assembled into a single contig and was circularized manually by deleting the overlapping end. The draft assembly was subsequently error corrected with three rounds of Pilon version 1.23 (8) polishing using 58.6 million raw Illumina short reads. Genome completeness was assessed using Benchmarking Universal Single-Copy Orthologs (BUSCO) version 1 (9) with the bacterial data set, using the gVolante Web server (10), resulting in 100% coverage of 40 BUSCO genes. Genes were annotated using the DDBJ Fast Annotation and Submission Tool (DFAST) pipeline (11), and the genome was rotated according to the location of the *dnaA* gene. The genome size is 2,371,107 bp, with a G+C content of 60.3%, containing 1,941 putative coding sequences, 8 rRNAs, 56 tRNAs, and 1 CRISPR region. Default parameters were used for all software unless otherwise specified.

A previous study showed that the *tad* gene cluster is essential for colonization of Bifidobacterium breve UCC2003 in the human gut microbiota (12). Our analysis with BLAST+ version 2.2.30 found all *tad* gene clusters reported in previous studies. Other than *tadV*, these genes are clustered within the genome, syntenic to that in the previous study. In this respect, the availability of a new genomic resource will facilitate studies on host colonization by bifidobacteria.

This study was approved by the ethics committee of Keio University Shonan Fujisawa Campus under approval number 195. The subject was informed of the purpose of this study, and written consent was obtained from the subject.

### Data availability.

The complete genome sequence of B. longum strain Jih1 has been deposited in DDBJ under accession number AP022868 and in the Sequence Read Archive (SRA) under BioProject accession number PRJNA613014.
